# Supplementary material for: Sustained-input switches for transcription factors and microRNAs are central building blocks of eukaryotic gene circuits
Source: Genome Biol. 2013 Aug 23;14(8):R85. doi: 10.1186/gb-2013-14-8-r85 (PMC4054853; doi:10.1186/gb-2013-14-8-r85)
Supplement: Additional file 5 — HTML Browsable Motif Output. Zipped folder containing all WaRSwap and FANMOD motif output, viewable in a web browser. [file gb-2013-14-8-r85-S5.ZIP › HTML_browsable_motif_output/FANMOD_ath_tair9/sigs_fanmodm-2000.pvals.heatmaps.html/motif_id_6_000111002_tftype_ath_upstream_-3000_0.html]

```
BG_MODEL = FANMOD
MOTIF_ID = 6_000111002
TF_TYPE = ath
UPSTREAM = -3000_0


PVals
FN_0.2	FN_0.4	FN_0.6	FN_0.8
dg_60.genes	0.305	0.261	0.156	0.092
dg_70.genes	0.623	0.637	0.411	0.141
dg_80.genes	0	0	0	0

ZScores
FN_0.2	FN_0.4	FN_0.6	FN_0.8
dg_60.genes	0.682	1.021	1.355	1.417
dg_70.genes	0.057	0.331	0.679	1.03
dg_80.genes	2.347	2.247	1.621	1.046

StDevs
FN_0.2	FN_0.4	FN_0.6	FN_0.8
dg_60.genes	380.642	268.181	118.405	44.083
dg_70.genes	234.731	164.381	73.097	26.255
dg_80.genes	5.092	6.028	4.919	3.328
```
